# Gas Permeability of Mold during Freezing Process Alters the Pore Distribution of Gelatin Sponge and Its Bone-Forming Ability

**DOI:** 10.3390/ma13214705

**Published:** 2020-10-22

**Authors:** Xiaoyu Han, Yoshitomo Honda, Tomonari Tanaka, Kazuki Imura, Yoshiya Hashimoto, Kazushi Yoshikawa, Kazuyo Yamamoto

**Affiliations:** 1 Department of Operative Dentistry, Osaka Dental University, Osaka 573-1121, Japan; hanxy9308@gmail.com (X.H.); imura@cc.osaka-dent.ac.jp (K.I.); kazushi@cc.osaka-dent.ac.jp (K.Y.); yamamoto@cc.osaka-dent.ac.jp (K.Y.); 2 Institute of Dental Research, Osaka Dental University, Osaka 573-1121, Japan; 3 Graduate School of Science and Technology, Kyoto Institute of Technology, Kyoto 606-8585, Japan; t-tanaka@kit.ac.jp; 4 Department of Biomaterials, Osaka Dental University, Osaka 573-1121, Japan; yoshiya@cc.osaka-dent.ac.jp

**Keywords:** gas permeability, lyophilization, pore distribution, gelatin, bone formation, freeze-dry

## Abstract

Freeze-drying, also known as lyophilization, is widely used in the preparation of porous biomaterials. Nevertheless, limited information is known regarding the effect of gas permeability on molds to obtain porous materials. We demonstrated that the different levels of gas permeability of molds remarkably altered the pore distribution of prepared gelatin sponges and distinct bone formation at critical-sized bone defects of the rat calvaria. Three types of molds were prepared: silicon tube (ST), which has high gas permeability; ST covered with polyvinylidene chloride (PVDC) film, which has low gas permeability, at the lateral side (STPL); and ST covered with PVDC at both the lateral and bottom sides (STPLB). The cross sections or curved surfaces of the sponges were evaluated using scanning electron microscopy and quantitative image analysis. The gelatin sponge prepared using ST mold demonstrated wider pore size and spatial distribution and larger average pore diameter (149.2 µm) compared with that prepared using STPL and STPLB. The sponges using ST demonstrated significantly poor bone formation and bone mineral density after 3 weeks. The results suggest that the gas permeability of molds critically alters the pore size and spatial pore distribution of prepared sponges during the freeze-drying process, which probably causes distinct bone formation.

## 1. Introduction

Three-dimensional porous materials have been widely used in various fields, such as regenerative medicine, as scaffolds [1], cell-seeding materials [2,3], or drug carriers [4]. Suitable porous structures of such materials contribute to the efficient supply of oxygen and nutrients [5], which significantly influence cell activity, such as vascular and cell ingrowth [6], and cellular differentiation [7,8], resulting in sufficient tissue regeneration [6]. Consequently, numerous research groups have addressed the improvement of pore size, pore alignment, and the porosity of various materials, including polymers and combinations of inorganic materials [7,8,9,10]. However, a further detailed examination of the freeze-drying technique may be required to verify its effect on pore distribution in prepared porous materials.

Freeze-drying, which is also known as lyophilization, has been used for a long time in the food, cosmetic, and pharmaceutical industries as well as in biotechnology, chemical synthesis, and regenerative medicine [2,4,11,12,13]. Freeze-drying is a process that involves removing water from a sample without destroying its three-dimensional structure. It comprises four steps: preparation of samples in the liquid state, prefreezing, freeze-drying during primary drying at sublimation, and secondary drying to remove the residual water [11]. At the prefreezing step, water in the samples is transformed into ice through water solidification [2,14]. Thus far, it has been the consensus that a porous structure can be modified by altering the cooling rate [4,12], freezing temperature [4,8,12,15], freezing process [10,16], annealing process [15], porogenic template [17], and/or concentration of samples [4]. The freezing temperature is thought to regulate pore size [15].

Gelatin, which is denatured collagen, is commonly obtained from the connective tissue of a murine source, pig, and bovine [18,19,20]. It demonstrates high biocompatibility, biodegradability, cost-effectiveness, sol–gel transition, and formability [21,22,23]. As with collagen, the stability of gelatin can be improved by crosslinking using the chemical [24] and physical methods [13]. This denatured protein is relatively easier to use than collagen. Therefore, it is used in various biomedical applications, including bone regenerative medicine [13,25]. The porous gelatin sponge is used alone [1] or in combination with other polymers [26], polyphenols [13,25], small molecules [4], or calcium phosphates [9] as a bone substitute material [9,13,25] or cell-seeding scaffold [3] in bone regenerative medicine. Thus far, various researchers have reported valuable findings for regulating the pore structure of gelatin sponges by altering the freezing temperature, freezing time, or sample concentration [4,10]. However, there is a paucity of information regarding the effect of gas permeability on molds for preparing pore distributions.

To this end, this study was undertaken to investigate whether different gas permeability levels of molds affect the pore structure of a freeze-dried gelatin scaffold and its bone-forming ability. Three different types of molds were used to alter the gas permeability in molds: (1) silicon tube with high permeability (ST); (2) ST covered with polyvinylidene chloride (PVDC), which has less permeability than ST, at the lateral side (STPL); and (3) ST covered with PVDC at the lateral and bottom sides (STPLB). The critical-sized bone defect in rat calvaria was adopted as an experimental animal model to evaluate the bone-forming ability of each type of gelatin sponge.

## 2. Materials and Methods

### 2.1. Materials and Molds

**Figure 1:**
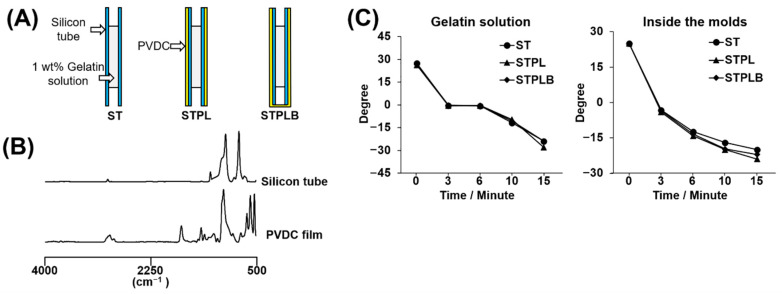
(**A**) Schematic images of three types of molds. ST: silicon tube showing high permeability; STPL: ST covered with polyvinylidene chloride (PVDC) at the lateral side; STPLB: ST covered with PVDC at the lateral and bottom sides. (**B**) Spectra of attenuated total reflection–Fourier transform infrared (ATR–FTIR) analysis on the silicon tube and the PVDC-contained film. (**C**) Temperature variation of gelatin solutions in molds or temperature variation inside of molds during prefreezing.

Medical-grade type A gelatin RM-100 derived from porcine skin and containing low levels of lipopolysaccharide (LPS) was purchased from Jellice (Miyagi, Japan). The ST (Φ5 mm × 8 cm; AS ONE, Osaka, Japan) and the polymer primarily composed of PVDC (designated as PVDC film; Saran Wrap, ASAHI KASEI, Tokyo, Japan) were used to prepare the three types of molds mentioned above, as shown in Figure 1A. Each material has been reported to show different levels of gas permeability. The silicon tube has high gas permeability, and the PVDC film has low gas permeability [27].

### 2.2. Preparation of Gelatin Sponges

To prepare the gelatin sponges, the gelatin water solution was initially prepared by dissolving 100 mg gelatin powder in 10 mL ultrapure water at 70 °C. The gelatin solution was stored at −20 °C before use. After melting in a water bath at 37 °C, 700 μL of the gelatin solution was injected into molds. Next, prefreezing at −30 °C for 3 h was carried out using a freezer (MDF-U442, SANYO Electric Co., Ltd., Osaka, Japan), after which freeze-drying for two days using DC800 (Yamato Co., Ltd., Tokyo, Japan) was performed. The obtained sponges were finally vacuum-heated using ETTAS AVO-250NS (AS ONE, Osaka, Japan) at 150 °C and at a gauge pressure of −0.1 MPa for 24 h to facilitate physical crosslinking. The macroscopic images of the vacuum-heated gelatin sponges were obtained using a Fujifilm X-T100 digital camera (FUJIFILM, Tokyo, Japan). The temperature change inside the molds and the temperature change of the gelatin solutions in the molds were also measured using a temperature sensor (Custom digital thermometer CT-220, Custom, Tokyo, Japan) in a −30 °C freezer.

### 2.3. Characterizations of Sponge Scaffolds and Materials

The morphologies of the sponges were evaluated using the S-4800 field emission scanning electron microscope (FE-SEM) (Hitachi, Tokyo, Japan). The upper and lower parts of the sponges were sectioned using a microtome blade to obtain the horizontal and vertical sections for observation. The sections and curved surfaces were observed after OsO_4_ coating using the HPC-20 device (Vacuum Device, Ibaraki, Japan). The SEM images were analyzed using ImageJ software (ImageJ 1.52v; NIH, Bethesda, MD, USA) to obtain the quantitative data of pore size, pore area, and circularity [28]. The mean value for each section image was recorded, and four low magnification images were evaluated. The sponges, ST, and PVDC film were evaluated using Fourier transform infrared spectroscopy (IRAffinity-1S, Shimadzu Corporation, Kyoto, Japan).

### 2.4. Hydrophilicity of the Sponges

The hydrophilicity of the sponges was evaluated by dropping 0.7 μL of ultrapure water on the cross sections and curved surfaces of the sponges. The time spent during water absorption was measured using a SEIKO ACRP 88-5061-1/10 s handheld stopwatch (Seiko Holdings Corporation, Tokyo, Japan). If the water was still not completely absorbed after 5 min, the absorption time was recorded as 300 s. The images at 5 s after water dropping were also taken under the magnified field of the contact angle meter LSE-ME (NiCK Corporation, Saitama, Japan). The contact angle was measured using ImageJ software (ImageJ 1.52v; NIH, Bethesda, MD, USA). Four samples of each type of sponges were tested.

### 2.5. Lipopolysaccharide Content Measurement

The sponges were dissolved in phosphate-buffered saline (PBS) at 10 mg/mL and ground using the Mixer Mill MM 301 (Verder Scientific, Haan, Germany). The LPS contents of the sponge solutions were assessed using the ToxinSensor Chromogenic LAL Endotoxin Assay Kit (GenScript Biotech Corporation, Piscataway, NJ, USA) according to the manufacturer’s instructions.

### 2.6. Cytotoxicity of the Sponges

The rat osteoblastic cell line UMR106 (ECACC, Public Health England, London, UK) was used to measure sponge cytotoxicity. UMR106 cells passage 4 were seeded at a density of 1 × 10^4^/ well and cultured in Dulbecco’s modified Eagle’s medium (Nacalai Tesque, Kyoto, Japan) with 10% fetal bovine serum and 1% antibiotics. One day after seeding, the medium was renewed and 0.5 mg of each type of sponges was added. The cells treated without sponges were also prepared as a control group. One or three days after the treatment with sponges, cell viability was evaluated using the Cell Counting Kit-8 (Dojindo Laboratories, Kumamoto, Japan) and the LIVE/DEAD™ Viability/Cytotoxicity Kit for mammalian cells L3224 (Molecular Probes, Eugene, OR, USA). The data from the Cell Counting Kit-8 were obtained using a plate reader, SpectraMax M5 (Molecular Devices, San Jose, CA, USA), and the live/dead fluorescent stain using the ZOE Fluorescent Cell Imager (Bio-Rad Laboratories, Hercules, CA, USA).

### 2.7. Animal Experiments

The animal experiments were approved by the Animal Experiment Committee of Osaka Dental University and strictly conformed to the guidelines (Approval No. 19-05001; approval date: 2019.6.6). Eight-week-old male Sprague–Dawley rats (Shimizu Laboratory Supplies, Kyoto, Japan) were anesthetized through the intraperitoneal injection of a mixture of butorphanol tartrate, midazolam, and medetomidine hydrochloride. Under cooling using sterile saline, critical-sized bone defects at the center of calvarias were created by utilizing a 9 mm diameter trephine bar (Dentech, Tokyo, Japan). One sponge (approximately 7 mg) was sliced and implanted in the bone defect. With different implants, the animals were divided into four groups (three rats per group): ST: the rats treated with gelatin sponges prepared using ST; STPL: those with STPL; STPLB: those with STPLB; Control: those without gelatin sponges. Three weeks after surgery, all the rats were euthanized. The calvarias were harvested and soaked in 4% paraformaldehyde phosphate buffer solution (FUJIFILM Wako Pure Chemical Corporation, Osaka, Japan) for further experiments.

### 2.8. Hematoxylin–Eosin Staining

All samples were decalcified using the decalcifying solution A (FUJIFILM Wako Pure Chemical Corporation, Osaka, Japan). The decalcified samples were then dehydrated and embedded in paraffin. Paraffin sections (3 μm in thickness) were prepared and stained with hematoxylin–eosin (H–E).

### 2.9. Bone Morphometry Using Microcomputed Tomography

Bone formation and morphometry were evaluated using microcomputed tomography (μCT) (SkyScan 1275, Bruker Corporation, Billerica, MA, USA). The fixed calvaria samples were scanned at 75 kV and 85 μA. The quantitative data of the morphometric parameters were then measured using the SkyScan™ CT analyzer (version 1.17.7.2) software.

### 2.10. Statistical Analysis

The results of all groups were evaluated using GraphPad Prism 8 (GraphPad Software Inc., San Diego, CA, USA) with a one-way analysis of variance (ANOVA), followed by the Tukey–Kramer test to evaluate the statistical significance between each group.

## 3. Results

### 3.1. Characterization of Molds

Figure 1A,B represents the schematic view of three different types of molds and the ATR–FTIR spectra of used silicon tube and PVDC film. The spectra of the silicon tube and PVDC film are coincident with those reported previously [29,30]. When the temperature change inside the molds and the temperature change of the gelatin solutions in the molds were analyzed, negligible differences were observed among the three types of molds (Figure 1C), suggesting that all types of molds provided similar thermal environments for the gelatin solutions during the prefreezing process.

### 3.2. Macroscopic and Scanning Electron Microscopic View of Gelatin Sponges

**Figure 2:**
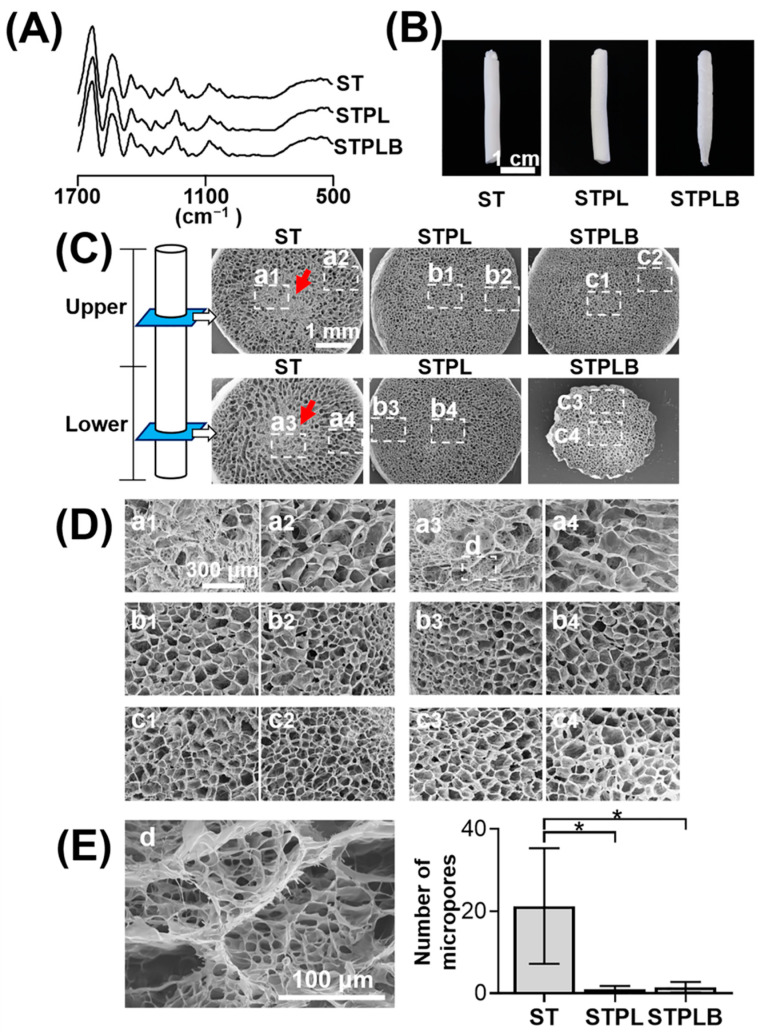
Characteristic of gelatin sponges prepared using different molds. ST: silicon tube; STPL: ST covered with PVDC at the lateral side; STPLB: ST covered with PVDC at the lateral and bottom sides. (**A**) ATR–FTIR spectra of gelatin sponges, (**B**) macroscopic images of gelatin sponges, (**C**) field emission scanning electron microscope (FE-SEM) images of cross-sectioned sponges with low magnification. Red arrows: dense structure in the center of gelatin sponges prepared using ST. a1–c4: magnified area for D. (**D**) Magnified SEM images of cross-sectioned sponges. d: magnified area for E. (**E**) Magnified SEM image of the central part of gelatin sponges prepared using ST. * *p* < 0.05, *n* = 4, one-way ANOVA with Tukey-Kramer tests.

Figure 2A shows the ATR–FTIR spectra of the sponges. No obvious changes were observed even after freeze-drying and dehydrothermal treatment in all sponges. Meanwhile, in comparison with the other two types of sponges, gelatin sponges prepared using STPLB exhibited a tapered shape at the bottom part of the sponges (Figure 2B). Figure 2C–E and Figure S1 show the horizontal and vertical SEM views of the sectioned sponges at the upper or lower parts. Small pores intensively aggregated at the center of the gelatin sponges prepared using ST (a1 and a3 of Figure 2C or Figure 2D; red arrows), whereas uniform pores were distributed in those prepared using STPL and STPLB (Figure 2C,D). In contrast to the sponges prepared using STPL and STPLB, small pores of approximately 10 µm could be observed among the large pores of the sponges prepared using ST (Figure 2E).

**Figure 3:**
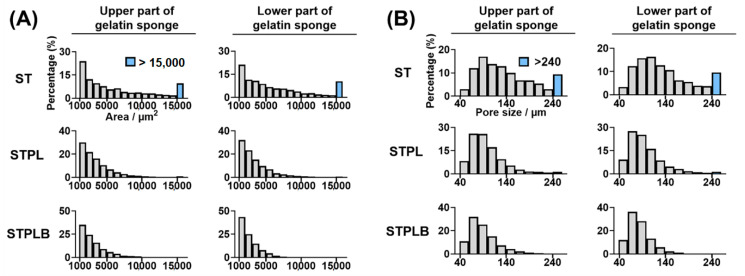
Distribution of pore area (**A**) and size (**B**) in horizontally sectioned gelatin sponges prepared using different molds. The data were obtained through quantitative image analysis using ImageJ software. ST: silicon tube showing high permeability; STPL: ST covered with PVDC at the lateral side; STPLB: ST covered with PVDC at the lateral and bottom sides. Representative data from four different images.

**Figure 4:**
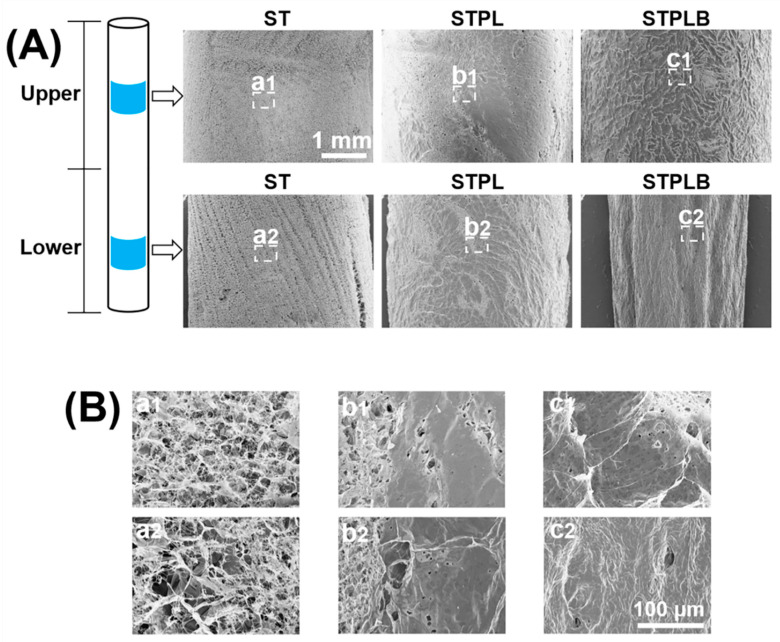
(**A**) Low and (**B**) high magnification of SEM images of the curved surfaces of gelatin sponges prepared using different molds. a1–c2: magnified images for B. ST: silicon tube showing high permeability; STPL: ST covered with PVDC at the lateral side; STPLB: ST covered with PVDC at the lateral and bottom sides.

The quantitative image analysis of pore size and area revealed that the range of the mean pore size of the sponges was between 87.1 and 149.2 μm (Table 1). Compared with other sponges, those prepared using ST had larger pores (Table 1 and Table S1) and a relatively wider distribution of pore area and size (Figure 3 and Figure S2). The sponges prepared using STPL and STPLB had higher circularity than those prepared using ST (Table 1 and Table S1). The curved surfaces of the sponges prepared using ST exhibited a porous structure for all surfaces, whereas those using STPL and STPLB contained smooth surfaces (Figure 4).

### 3.3. Effect of Mold during Freezing or Drying

**Figure 5:**
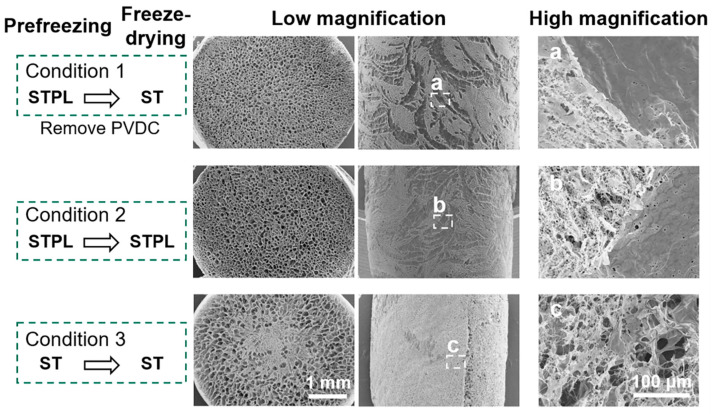
Evaluating the effect of gas permeability on molds during the freezing or drying process. SEM images: cross sections and curved surfaces of gelatin sponges prepared under different conditions. Condition 1: STPL→ST (prefreezing in the STPL mold, followed by removing PVDC during the freeze-drying process); condition 2: STPL→STPL (prefreezing and freeze-drying in the STPL mold); condition 3: ST→ST (prefreezing and freeze-drying in the ST mold).

In an attempt to evaluate whether different molds affect at the course of prefreezing or the freeze-drying process, gelatin sponges were prepared under the condition that PVDC was removed from STPL after the prefreezing process (condition 1) (Figure 5). The gelatin sponges prepared under condition 1 retained similar inner and surface pore structures in comparison with the sponges prepared under condition 2 (without removing PVDC during the freeze-drying process). Additionally, they differed from the sponges prepared under condition 3, suggesting that the change in gas permeability primarily influenced the prefreezing process and not the freeze-drying process.

### 3.4. Water Absorption Capacity of Sponges

**Figure 6:**
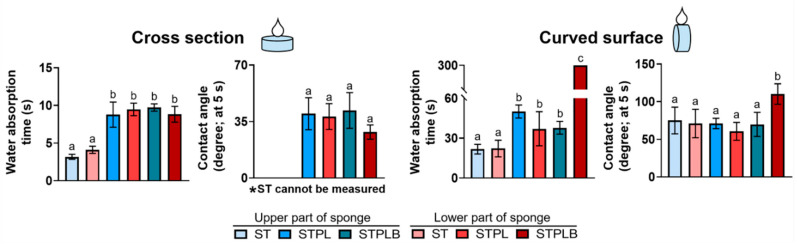
Water absorption time and contact angles at the cross sections or curved surfaces of the gelatin sponges prepared using the different molds. ST: silicon tube showing high permeability; STPL: ST covered with PVDC at the lateral side; STPLB: ST covered with PVDC at the lateral and bottom sides. Same alphabet: no statistical significance. Mean with SD (*n* = 4, *p* < 0.05, one-way ANOVA with Tukey–Kramer tests).

The water absorption capacity of sponges is considered to affect blood absorption, which has the potential to alter the bone-forming ability [31]. When testing the water absorption capacity of sponges using their cross sections and curved surfaces, the upper and lower parts of the gelatin sponges prepared using ST with larger pores demonstrated faster water absorption rates than those of the gelatin sponges prepared using STPL and STPLB (Figure 6 and Figure S3). Contact angle measurement revealed that at 5 s, the cross sections of the sponges showed higher hydrophilicity than that of the curved surfaces. However, negligible differences were observed among the curved surfaces of the sponges (Figure 6).

### 3.5. Measurement of Lipopolysaccharide Contamination and Cytotoxicity on Sponges

**Figure 7:**
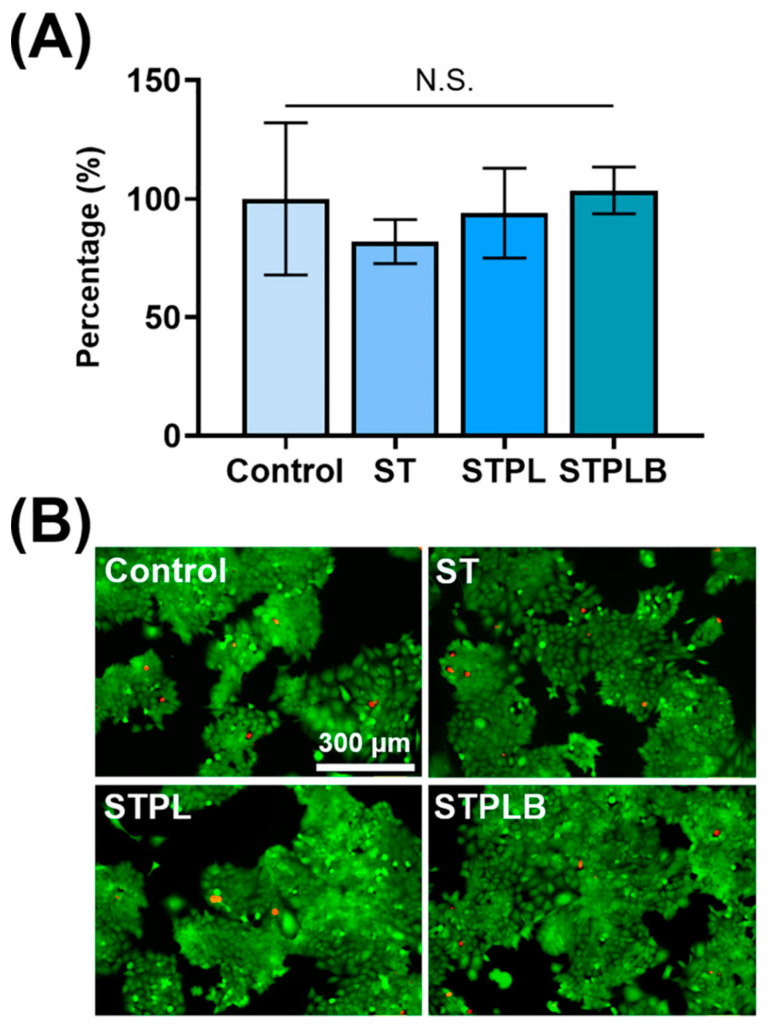
Evaluation of cytotoxicity in vitro at day 3. Rat osteoblastic cell line UMR106 cells treated with gelatin sponges prepared using three different molds: ST: silicon tube; STPL: ST covered with PVDC at the lateral side; STPLB: ST covered with PVDC at the lateral and bottom sides. Control: no sponges. (**A**) WST-8 assay. Mean with SD (*n* = 4, *p* > 0.05, one-way ANOVA with Tukey–Kramer tests). N.S.: no statistical difference. (**B**) Live or dead viability staining. Green: live cells; red: dead cells.

Using the Endotoxin Assay Kit, we evaluated the LPS level in sponges prepared using ST, STPL, or STPLB. The LPS levels in each sponge were 0.017 ± 0.006 EU/mg for ST, 0.018 ± 0.008 EU/mg for STPL, and 0.017 ± 0.003 EU/mg for STPLB, which still retained low levels similar to the intrinsic medical-grade gelatin even after the synthesis process. All three sponges had negligible cytotoxic effects on the rat osteoblastic cell line UMR106 cells after 1 or 3 days of treatment (Figure 7 and Figure S4).

### 3.6. Bone-Forming Ability of Sponges

**Figure 8:**
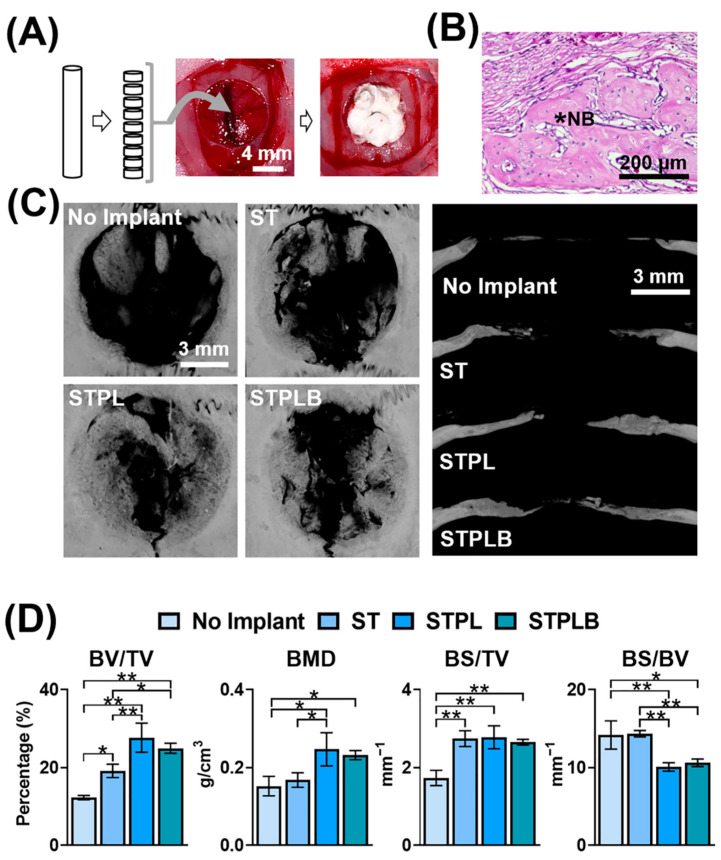
(**A**) Schematic flow chart of the animal experiments. Sliced gelatin sponges were implanted in critical-sized bone defects of rat calvaria. (**B**) Representative hematoxylin–eosin staining image of the defects (the defect treated with the gelatin sponges prepared using STPL is presented). *NB: newly formed bone. (**C**) Vertical and lateral microcomputed tomography (μCT) images of bone defects treated with/without gelatin sponges prepared using different molds. Three weeks after surgery. ST: silicon tube showing high permeability; STPL: ST covered with PVDC at the lateral side; STPLB: ST covered with PVDC at the lateral and bottom sides. (**D**) Bone morphometry analysis of newly formed bone using μCT. Mean with SD (*n* = 3). BV, bone volume; TV, total volume; BMD, bone mineral density; BS, bone surface. * *p* < 0.05, ** *p* < 0.01, one-way ANOVA with Tukey–Kramer tests.

The dissected sponges were implanted in the bone defects for 3 weeks to estimate the bone-forming ability of each sponge using histological and µCT evaluation (Figure 8 and Figure S5). Even after 3 weeks, a newly formed bone (NB) could be observed at all defects under histological evaluation (Figure 8B and Figure S5), which was consistent with the radiopacity site in the bone defect evaluated using µCT analysis (Figure 8C). Although the sponges prepared using the ST, STPL, and STPLB molds increased bone formation in comparison with the group with no implant, those prepared using ST demonstrated less bone formation (BV/TV) and bone mineral density (BMD) than the sponges prepared using the other two types of molds (Figure 8D).

## 4. Discussion

Our results indicate that the gas permeability of molds during the freezing process significantly regulates the pore size and spatial pore distribution of the obtained gelatin sponges. These structural differences remarkably affected the bone-forming ability in critical-sized bone defects of rat calvaria.

The freezing temperature is a modulator for altering the porous structure of sponges [4,8,12,15]. In view of the different thermal conductivity of ST and PVDC, we used ST as the inner part of all the molds. These STs were covered with PVDC for STPLs and STPLBs instead of using PVDC alone for the molds. Therefore, all the gelatin solution directly contacted ST and not PVDC. The interfaces between the gelatin solution and the molds were consistent in three different types of molds. In fact, there was negligible difference in temperature change between the gelatin solutions and the inner parts of the molds (Figure 1C). In contrast, it is known that water typically traps dissolved air [32,33]. The dissolved air is presumed to be dischargeable from ice [33,34] during ice solidification as a result of the absence of space in ice lattices [34]. This principle is occasionally noted to prepare clear ice in the industry. STs have higher gas permeability than STPLs and STPLBs because they lack PVDC, suggesting that the dissolved air in the gelatin solution might have been able to escape through ST during the freezing process. In fact, from our data, the gelatin sponges prepared using ST presented a highly porous structure at the curved surfaces in comparison with those prepared using STPL or STPLB (Figure 4). No remarkable changes could be observed if PVDC was removed from STPL before the drying process (Figure 5). Although the detailed migration of air during the prefreezing process could not be tracked, given these results, distinct air migration or residue attributed to the different molds may partially affect the pore distribution of the prepared gelatin sponges.

LPS, which is an outer-cell membrane of Gram-negative bacteria, is an established strong stimulant for modulating the immune system and inducing inflammation [35,36], which diverges bone formation that is primarily dependent on its dose and sustained condition to the matrix materials [36,37,38]. We have previously reported that even tiny contaminations involving minute amounts of LPS in gelatin sponges remarkably hindered bone formation in a similar bone defect model [38]. Xu et al. also reported that the combined use of the NF-κB activator and gelatin augmented severe inflammation [39]. In view of these backgrounds, we evaluated LPS contamination and the cytotoxicity of the prepared gelatin sponges before the in vivo test. All sponges showed similarly low levels (extremely low levels) of LPS and no cytotoxicity for the cells in vitro, suggesting that the distinct bone formation of the sponges might be partially due to the structural differences of the sponges.

Numerous studies have reported the effect of pore size on bone formation using the mean value as a scale [7,26,39,40]. Although the optimal mean pore sizes for bone regeneration using biodegradable polymers are still controversial, in inorganic substances, a pore size of over 100 µm is considered the preferable size [5,41]. In our study, the gelatin sponges prepared using ST had a larger mean pore size at the inside or the curved surfaces in comparison with those prepared using STPL and STPLB. Furthermore, the large pores presumably contribute to the alteration of the material property of the sponges at least when considering the water absorption result (Figure 6). Nevertheless, the gelatin sponges prepared using ST exhibited obvious lower bone formation (BV/TV) and BMD in comparison with those prepared using STPL (Figure 8). The sponges prepared using ST demonstrated a considerably heterogeneous distribution of size and space compared with those prepared using STPL and STPLB. Both STPL and STPLB contributed to the preparation of similar uniform pore structures. Given these results, the distinct pore distribution attributed to the gas permeability of molds is associated with the differences in bone-forming speed. At an appropriate size, the uniformity of pores and the large mean pore size may play an important role in the promotion of the bone-forming ability of sponges.

We have proven that the gas permeability of molds alters the pore distribution of gelatin sponges, resulting in distinct bone formation. Previous studies have reported that small pores (smaller than 75 μm) impair osteoconduction [41,42]. The poor bone formation in defects treated with gelatin sponges prepared using ST might be partially caused by the concentrative localization of small pores (approximately 10 μm) at the center of the sponges. In fact, there appears to be poor bone formation at the center of defects treated with sponges prepared using ST compared with other defects treated with sponges using STPL and STPLB. However, the exact mechanism underlying how gas permeability diverges the pore distribution and how pore distribution affects bone formation remains unclear. We have not regulated the detailed level of gas permeability of molds. It is still unclear whether other molds represent similar pore structures for gelatin sponges. We may need to clarify the behavior of frozen gelatin sponges in detail. Because image analyses were used, instead of gas or mercury porosimetry, for evaluating pore structures and spatial pore distribution at the small scale, we failed to obtain data related to meso- and micropores. Furthermore, exploring optimal pore distribution for bone formation might provide valuable insight into bone regeneration and bone biology. Given these limitations, although further examination would be essential, our results provide an insight into the development of novel porous scaffolds for bone regeneration using biodegradable polymers.

## 5. Conclusions

This study demonstrated that pore size and spatial pore distribution are altered by the gas permeability of molds. During the prefreezing process, the increase in the gas permeability of molds enhanced the heterogeneity of pore size and the spatial distribution of pores in the obtained gelatin sponges. Meanwhile, the gelatin sponges prepared using a polymer (PVDC) with lower gas permeability in the lateral and bottom sides yielded more uniform pores. The difference significantly affected the bone-forming ability of sponges. These results indicate that the gas permeability of molds must be cautiously taken into account during the prefreezing process at the lyophilization for preparing optimal porous materials and gelatin-based materials. Additionally, attention must be paid to the pore size distribution and spatial pore distribution even when using biomaterials produced clinically.

## Figures and Tables

**Table 1:** Mean size and circularity of pores analyzed using SEM images of horizontally sectioned gelatin sponges.

	Upper Part of Sponge			Lower Part of Sponge		
	ST	STPL	STPLB	ST	STPL	STPLB
**Diameter (μm)**	149.2 a (10.9)	101.8 b (7.2)	93.0 b (1.8)	147.0 a (15.7)	100.6 b (6.5)	87.1 b (5.0)
**Circularity**	0.19 a (0.03)	0.31 b (0.03)	0.31 b (0.01)	0.20 a (0.02)	0.29 b (0.02)	0.34 b (0.02)

Numbers in parentheses: standard deviation. Same alphabet: no statistical difference. Mean: average of four regions of interest using SEM images of four different images.

## Supplementary Materials

The following are available online at https://www.mdpi.com/1996-1944/13/21/4705/s1: Figure S1: SEM images of vertically sectioned sponges, Figure S2: The distribution of pores in vertically sectioned gelatin sponges, Figure S3: Macro images of water absorption at sponges, Figure S4: Evaluation of cytotoxicity at day 1; Figure S5: Hematoxylin–eosin staining images, Table S1: Mean size and circularity of pores analyzed using SEM images of vertically sectioned gelatin sponges.
