# Supplementary material for: Gas Permeability of Mold during Freezing Process Alters the Pore Distribution of Gelatin Sponge and Its Bone-Forming Ability
Source: Materials (Basel). 2020 Oct 22;13(21):4705. doi: 10.3390/ma13214705 (PMC7659933; doi:10.3390/ma13214705)
Supplement: Supplementary file 1 [file materials-13-04705-s001.pdf]

materials

Supplementary File

Gas Permeability of Mold during Freezing Process Alters the Pore Distribution of Gelatin Sponge and Its Bone-Forming Ability

Xiaoyu Han [1], Yoshitomo Honda [2,*], Tomonari Tanaka [3], Kazuki Imura [1], Yoshiya Hashimoto [4], Kazushi Yoshikawa [1] and Kazuyo Yamamoto [1]

[1] Department of Operative Dentistry, Osaka Dental University, Osaka 573-1121, Japan; hanxy9308@gmail.com (X.H.); imura@cc.osaka-dent.ac.jp (K.I.); kazushi@cc.osaka-dent.ac.jp (K.Y.); yamamoto@cc.osaka-dent.ac.jp (K.Y.)

[2] Institute of Dental Research, Osaka Dental University, Osaka 573-1121, Japan

[3] Graduate School of Science and Technology, Kyoto Institute of Technology, Kyoto 606-8585, Japan; t-tanaka@kit.ac.jp

[4] Department of Biomaterials, Osaka Dental University, Osaka 573-1121, Japan; yoshiya@cc.osaka-dent.ac.jp

* Correspondence: honda-y@cc.osaka-dent.ac.jp; Tel.: +81-72-864-3130

Received: 6 October 2020; Accepted: 19 October 2020; Published: 22 October 2020

1. SEM Images of Vertically Sectioned Sponges

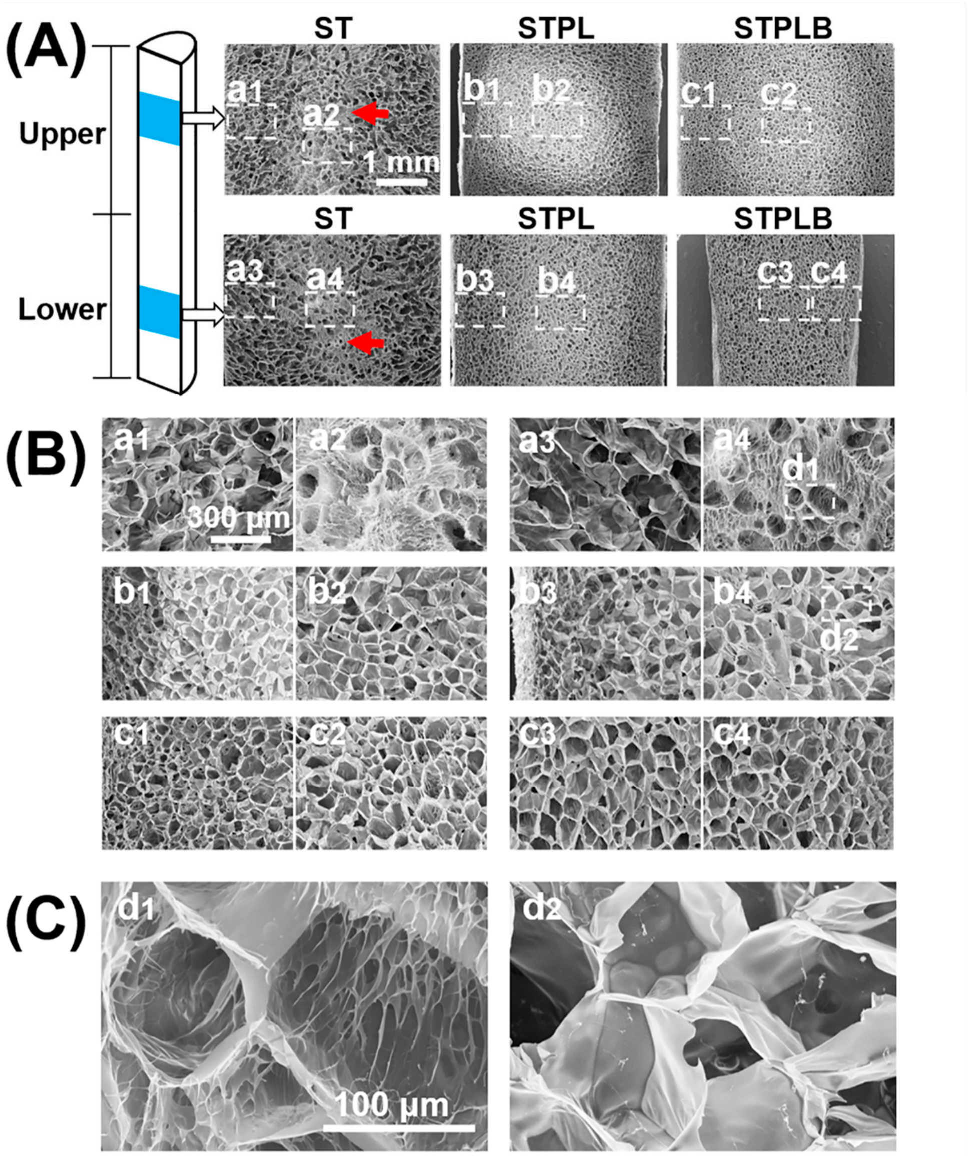

Figure S1. (**A**) Field-emission scanning electron microscope (FE-SEM) images of vertically sectioned sponges with low magnification. Red arrows: dense structure in the center of the gelatin sponges prepared using the ST. a1–c4: magnified area for B; (**B**) Magnified SEM images of vertically sectioned sponges. d1 and d2: magnified area for C; (**C**) Magnified SEM images of the central part of gelatin sponges prepared using the ST and STPL. ST: silicon tube showing high permeability; STPL: ST covered with polyvinylidene chloride (PVDC) at the lateral side; STPLB: ST covered with PVDC at the lateral and bottom sides.

Table S1. Mean size and circularity of pores analyzed using SEM images of vertically sectioned gelatin sponges.

	Upper Part of Sponge			Lower Part of Sponge		
	**ST**	**STPL**	**STPLB**	**ST**	**STPL**	**STPLB**
**Diameter (µm)**	152.8 a<br>(28.5)	107.7 b<br>(2.6)	89.9 b<br>(4.5)	151.2 a<br>(4.7)	106.6 b<br>(11.2)	90.2 b<br>(4.2)
**Circularity**	0.20 a<br>(0.02)	0.27 b<br>(0.02)	0.31 b<br>(0.02)	0.19 a<br>(0.01)	0.27 b<br>(0.01)	0.31 b<br>(0.03)

Numbers in parentheses: standard deviation. Same alphabet: no statistical difference. Mean: average of four regions of interest using SEM images of four different images.

2. The Distribution of Pores in Vertical Sectioned Gelatin Sponges

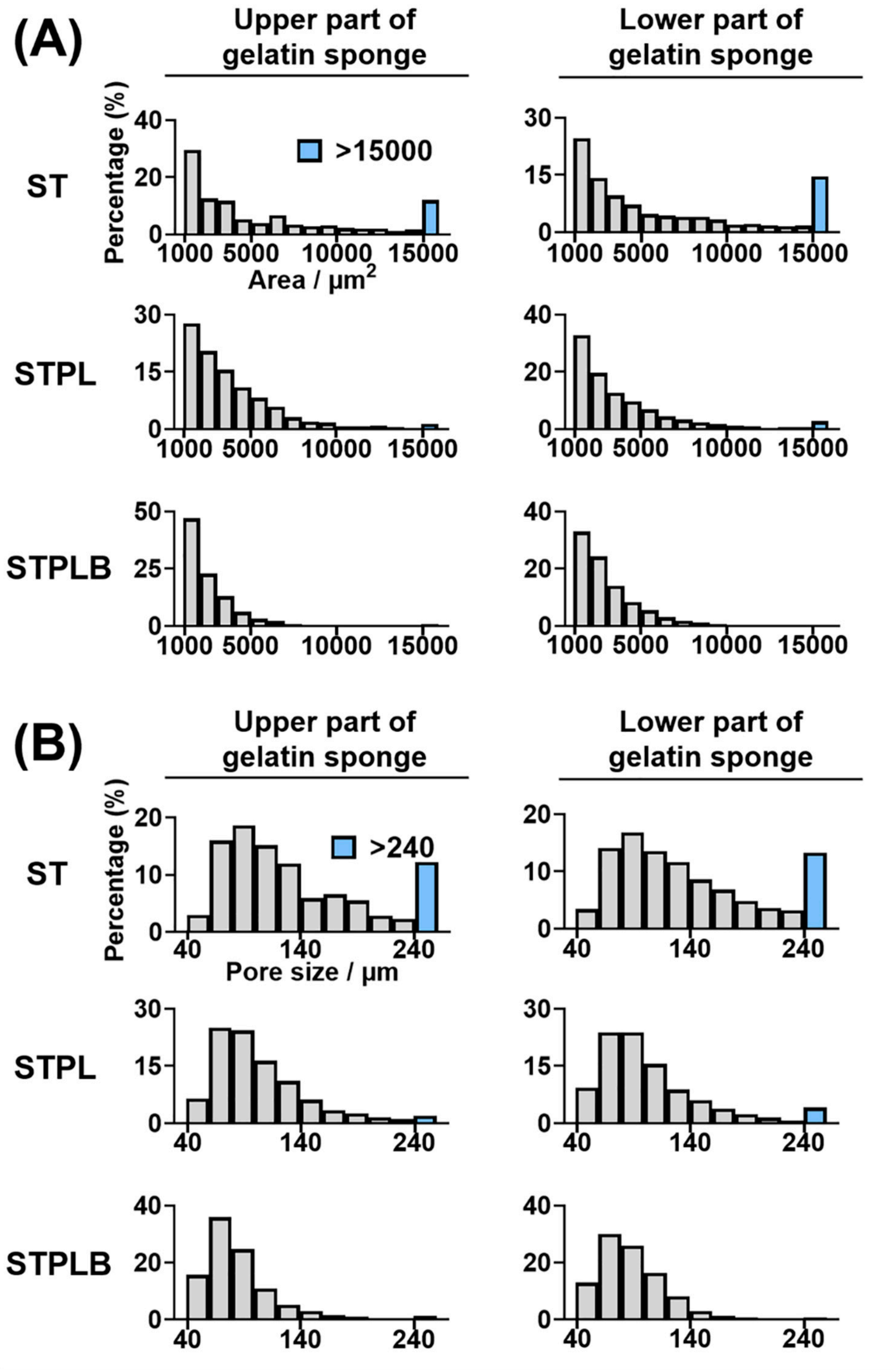

Figure S2. Distribution of pore area (**A**) and size (**B**) in vertically sectioned gelatin sponges prepared using different molds. The data were obtained through quantitative image analysis using Image J software. ST: silicon tube showing high permeability; STPL: ST covered with PVDC at the lateral side; STPLB: ST covered with PVDC at the lateral and bottom sides. Representative data from four different images.

3. Macro Images of Water Absorption at Sponges

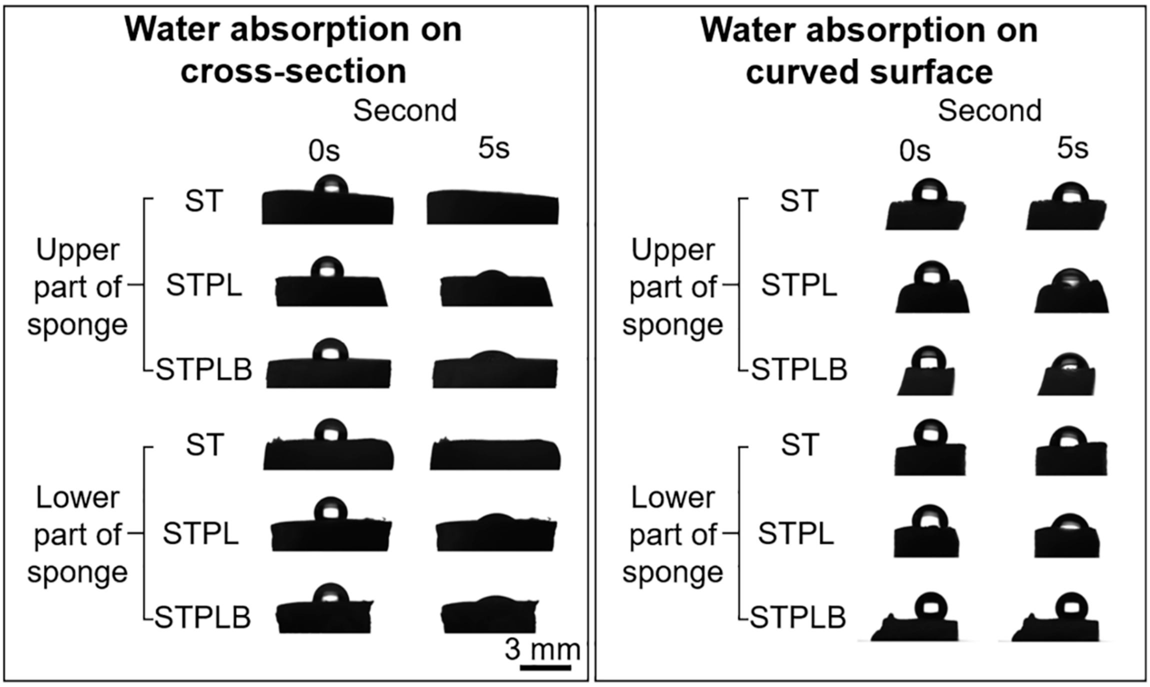

Figure S3. Macro images of water absorption at the cross-sections or curved surfaces of gelatin sponges prepared using the different molds. ST: silicon tube showing high permeability; STPL: ST covered with PVDC at the lateral side; STPLB: ST covered with PVDC at the lateral and bottom sides.

4. Evaluation of Cytotoxicity at Day 1

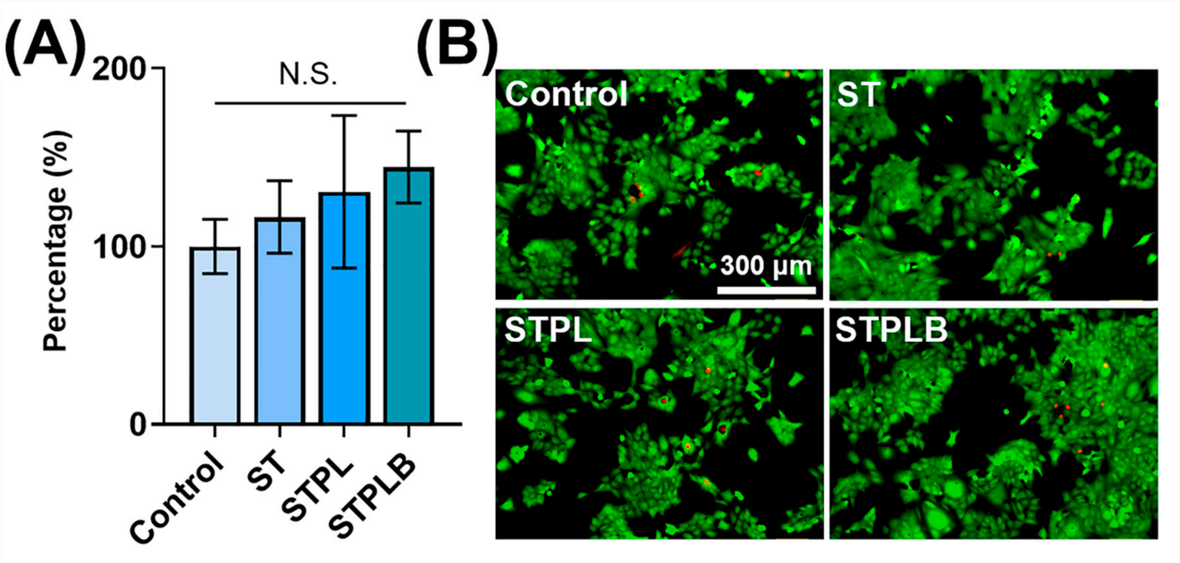

Figure S4. Evaluation of cytotoxicity *in vitro* at day 1. Rat osteoblastic cell line UMR106 cells treated with gelatin sponges prepared using three different molds: ST: silicon tube; STPL: ST covered with PVDC at the lateral side; STPLB: ST covered with PVDC at the lateral and bottom sides. Control: no sponges. (**A**) WST-8 assay. Mean with SD (n=4, $p > 0.05$, one-way ANOVA with Tukey-Kramer tests) N.S.: no statistical difference. (**B**) Live or dead viability staining. Green: live cells; red: dead cells.

5. Hematoxylin-Eosin Staining Images

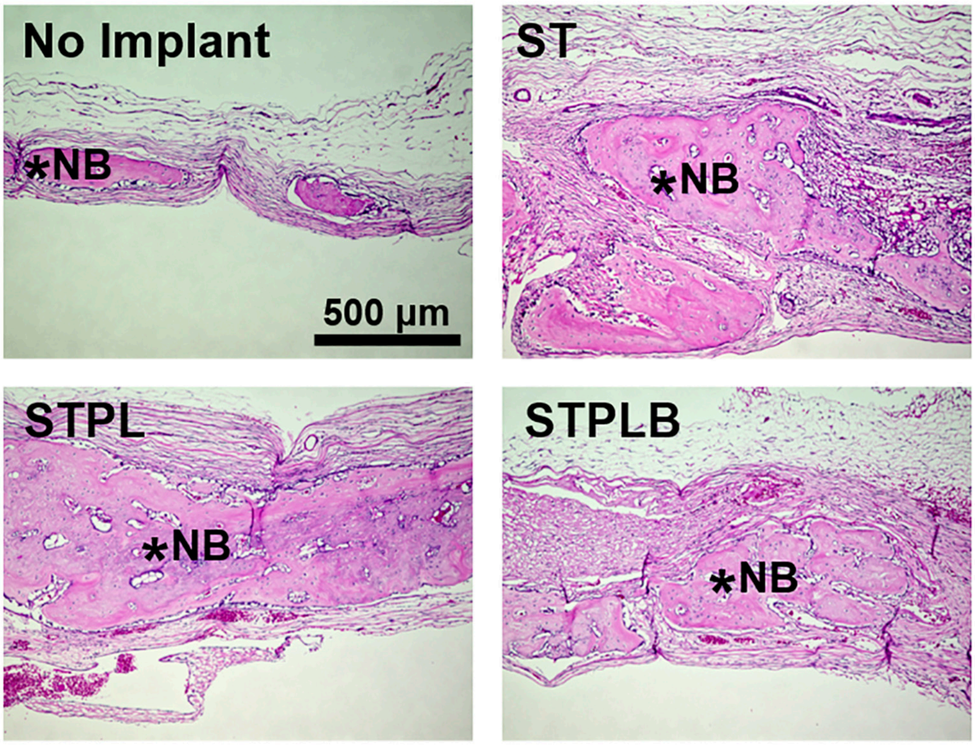

Figure S5. Representative hematoxylin-eosin staining images of the defects treated with/without gelatin sponges prepared using different molds. *NB: newly formed bone. ST: silicon tube; STPL: ST covered with PVDC at the lateral side; STPLB: ST covered with PVDC at the lateral and bottom sides.

Publisher's Note: MDPI stays neutral with regard to jurisdictional claims in published maps and institutional affiliations.

© 2020 by the authors. Licensee MDPI, Basel, Switzerland. This article is an open access article distributed under the terms and conditions of the Creative Commons Attribution (CC BY) license (http://creativecommons.org/licenses/by/4.0/).
